# A national survey on laboratory practices for specimen processing, streptococcus pneumoniae identification and antimicrobial susceptibility testing in CARSS Hospitals in China

**DOI:** 10.1371/journal.pone.0353484

**Published:** 2026-08-12

**Authors:** Cui Jian, Xueman Wang, Shaozhen Yan, Chao Xu

**Affiliations:** Department of Laboratoty Medicine, Tongji Hospital, Tongji Medical College, Huazhong University of Science and Technology, Wuhan, China; Children's National Hospital, George Washington University, UNITED STATES OF AMERICA

## Abstract

Standardized processing of respiratory specimens and accurate identification and antimicrobial susceptibility testing (AST) of Streptococcus pneumoniae are crucial for clinical management. This study investigated laboratory practices for specimen processing, S. pneumoniae identification, and AST of *S. pneumoniae*among CARSS hospitals. A nationwide online survey was conducted in 2023. After data cleaning, 3,297 valid questionnaires were analyzed. The survey assessed specimen distribution, sputum/BALF processing, identification and quality control (QC). Sputum was the most common specimen(39.92%), followed by “others” (20.83%), blood (20.47%), urine (14.16%), and BALF (4.58%). Most laboratories performed routine Gram staining on sputum (71.55%) and BALF (66.88%). For sputum smears, 75.80%  ~ 86.29% reported semi-quantitative cell/pathogen counts. The dominant acceptability criterion for sputum was > 25 WBCs/LPF and <10 SECs/LPF (98.88%). For unacceptable sputum specimens yielding S. pneumoniae 45.12% laboratories laboratories performed identification/AST only if the bacterial quantity was ≥ 2 + . Common identification methods were Optochin/bile solubility test (53.32%), Vitek GP card (42.74%) and MALDI-TOF MS (30.15%). AST methods included Vitek GP68 (38.76%), disk diffusion (32.91%), gradient diffusion (22.78%) and DL-96STREP cards (21.20%). Notably, 44.10% perform no routine AST QC and 44.62% lacked QC strain ATCC 49619. Only 49.65% used ATCC 49619 for QC.Significant variations exist in laboratory practices for respiratory specimen processing and S. pneumoniae workup across CARSS hospitals. While conventional methods remain prevalent, MALDI-TOF MS adoption is growing.The high proportion of laboratories lacking routine AST QC underscores the need for standardized protocols and enhanced quality assurance nationwide.

## Introduction

*Streptococcus pneumoniae* (also known as pneumococcus) is a Gram-positive, extracellular opportunistic pathogen that colonizes the mucosal surfaces of the human upper respiratory tract (URT). Between 27% and 65% of children and <10% of adults are carriers of *S. pneumoniae*. Carriage involves a symbiotic relationship between the bacterium and the host. Local spread, aspiration, or dissemination into the bloodstream can lead to invasive inflammatory diseases [1]. *S. pneumoniae* is a leading bacterial cause of a wide range of infections, including otitis media, community-acquired pneumonia, sepsis, and meningitis [2]. In 2021, *S. pneumoniae* was the leading pathogen globally among the 18 modelled categories, causing the most LRI episodes and deaths [3]. World Health Organization (WHO) listed S. pneumoniae as one of its 12 priority pathogens in 2017 andthe U.S. CDC categorized drug-resistant *S.pneumoniae* under the ‘serious threat’ level in 2013 and again in 2019 [4–6]. In the 2024 WHO updated bacterial priority pathogen list, *S.pneumoniae* is categorized as a medium-priority pathogen [4]. It is also a key monitoring indicator in the China Antimicrobial Resistance Surveillance System(CARSS) [7].

Accurate and timely identification and antimicrobial susceptibility testing (AST) of S. pneumoniae are critical for guiding appropriate antibiotic therapy and monitoring resistance trends. While lower respiratory tract specimens, especially sputum and bronchoalveolar lavage fluid (BALF), represent the most commonly submitted sample types in clinical practice, they also serve as a cornerstone for diagnosing lower respiratory tract infections.Standardized processing, including smear examination and culture, is essential for ensuring specimen quality and interpreting culture results [8]. Previous studies have highlighted variations in laboratory practices for microbiological diagnosis across different regions and healthcare settings in China [9]. Although molecular assays and MALDI-TOF MS(Matrix-Assisted Laser Desorption/Ionization Time-of-Flight Mass Spectrometry) are increasingly used for Streptococcus species identification, bacterial culture remains the gold standard for many clinical and surveillance purposes [10–15]. However, a comprehensive, recent national survey focusing specifically on practices related to lower respiratory tract specimen processing and S. pneumoniae workup in CARSS hospitals is lacking. Understanding current practices is the first step towards identifying areas for improvement and promoting standardization.

This national survey aimed to:(1) assess compliance with existing national and international guidelines for respiratory specimen processing and S. pneumoniae identification/AST; (2) benchmark current laboratory practices across CARSS hospitals to identify gaps and variability; and (3) provide evidence-based recommendations to standardize procedures, particularly for AST quality control. While we primarily describe the current landscape, the data also allow comparisons with recommended standards and highlight areas requiring urgent intervention.

The findings of this survey will serve as a critical baseline for future interventions and policy-making. By identifying specific deficiencies—such as the widespread lack of routine AST QC and the underutilization of the recommended QC strain ATCC 49619—this study provides actionable targets for quality improvement programs. Furthermore, the data inform the CARSS network on the current state of laboratory readiness, enabling targeted training and resource allocation. The methodological framework can also be adapted for similar surveys in other low- and middle-income countries.

Specifically, we sought to answer: (1) What are the current specimen type distributions and sputum/BALF processing practices in CARSS hospitals? (2) To what extent do laboratories adhere to recommended methods for S. pneumoniae identification and AST? (3) What proportion of laboratories perform routine AST quality control, and which QC strains are used?

## Methods

### Survey design and distribution

A cross-sectional online questionnaire survey was conducted among hospitals participating in the CARSS network in 2023. The questionnaire was designed to gather information on:

1. The proportional distribution of common specimen types (sputum, BALF, blood, urine, others) sent for bacterial culture annually.
2. Laboratory procedures for sputum and BALF smears (routine performance, content reported).
3. Criteria for assessing sputum and BALF quality.
4. Actions taken for unacceptable sputum specimens yielding S. pneumoniae.
5. Culture media used for lower respiratory tract specimens.
6. Inoculation methods for sputum and BALF.
7. Methods used for S. pneumoniae identification.
8. Brands and quality control practices for MALDI-TOF MS instruments.
9. Methods used for S. pneumoniae AST.
10. Frequency of AST quality control and the QC strains used.

The questionnaire comprised multiple-choice questions (single and multiple answer). It was distributed electronically to CARSS member hospitals. Participation was voluntary.

This survey was based on the CARSS data reporting system. All participants gave their informed consent before completing and submitting the questionnaire.

### Data collection and analysis

Data were collected from October 1 to October 15, 2023. A total of 3,526 initial responses were received. Data cleaning involved:

1. Removing 4 questionnaires lacking hospital names.
2. Identifying and handling duplicates: 228 questionnaires were identified as duplicates from 210 hospitals. For the 18 hospitals that submitted multiple identical responses, only one response was retained. For other duplicates, all but one were removed.
3. Removing 15 questionnaires based on post-sampling quality checks.

Finally, 3,297 valid questionnaires were included in the statistical analysis. Data analysis was performed using descriptive statistics. Categorical variables were presented as counts and percentages (n, %). Continuous variables (like specimen proportions) were summarized using averages and ranges. As this was an exploratory national survey without a priori hypotheses or comparator groups, only descriptive statistics (counts, percentages, averages, and ranges) were used. Inferential statistics were not performed because the study aimed to characterize the current landscape rather than test for differences between subgroups

## Results and discussion

### Response distribution and specimen type proportions

**Fig 1:**
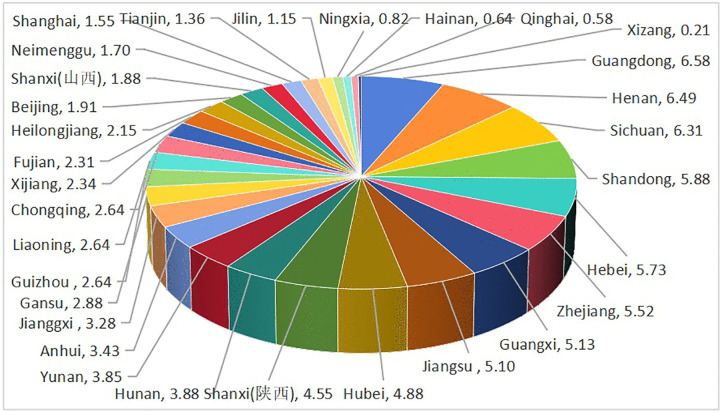
Geographical distribution of survey questionnaires across provinces in China (N = 3297).

Questionnaires were received from hospitals across all provinces in China. Guangdong, Henan, and Sichuan provinces contributed over 200 responses each; Shandong, Hebei, Zhejiang, Guangxi, Jiangsu, Hubei, and Shaanxi contributed 150–200 responses each; Hunan, Yunnan, Anhui, and Jiangxi contributed 100–149 responses each; other provinces contributed fewer than 100 responses (Fig 1).

**Fig 2:**
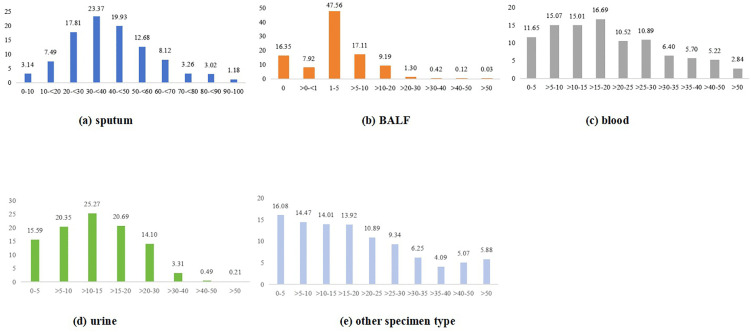
The distribution of different specimen types among annual bacterial culture submissions (N = 3297).

The average proportional distribution of different specimen types among annual clinical bacterial culture submissions is shown in Fig 2. Sputum constituted the largest share (39.92%, range 0–100%), followed by “other” specimens (20.83%, range 0–100%), blood (20.47%, range 0–100%), urine (14.16%, range 0–80%), and BALF (4.58%, range 0–53.2%).

### Sputum and BALF smear practices

71.55% (2359/3297) of laboratories performed Gram stain smears on sputum regardless of physician's order. 66.88% (2205/3297) performed Gram stain smears on BALF regardless of physician's order. For sputum smear content, 86.29% reported semi-quantitative WBC/SEC counts, and 75.80% reported presence/type of bacteria/pathogens.Pathogen type and semi-quantitative/quantitative counts were reported for 58.26% of laboratory submissions

#### Specimen acceptability criteria and handling of unacceptable sputum specimens.

The dominant criterion for acceptable sputum was > 25 WBCs/LPF and <10 SECs/LPF (98.88%).49.83% of laboratories performed acceptability screening for BALF. Among them, 55.29% used <1% SECs as the criterion, while 44.71% used the same criteria as for sputum.

For unacceptable sputum specimens containing *S. pneumoniae*, identification and antimicrobial susceptibility testing were performed in only 24.60% of cases (811 out of 3,297), regardless of the bacterial load.

#### *S. pneumoniae* identification methods.

The identification methods for *S.pneumoniae* are listed in Table 1. Approximately 50% of the laboratories did not routinely conduct the Optochin disc and/or bile solubility assays.Among the 623 laboratories utilizing MALDI-TOF MS platforms not manufactured by bioMérieux, 113 (18.14%) did not adhere to the supplementary identification procedures stipulated in the operating documentation.

**Table 1:** Proportion of kit/card/method identification of *S. pneumoniae.*

Identification kit/card/method	Proportion(%)
Optochin paper and/or bile solubility test	53.32
Vitek GP identification card^a^	42.74
MALDI-TOF MS^b^	30.15
DL Strept IST^c^	23.11
BD SMIC/ID-2^d^	8.07
Auto Gram-Positive Bacteria Identification Kit^e^	1.46
others	15.02

^a^bioMérieux company.

^b^mutiple MALDI-TOF MS brand including Bruke,bioMérieux,Antuo etc.

^c^DL Biotech company.

^d^Becton, Dickinson and Company.

^e^Autobio company.

#### *S. pneumoniae* AST methods.

The methodologies employed for the antimicrobial susceptibility testing of Streptococcus pneumoniae are depicted in Table 2. As per the specifications of the automated susceptibility testing instruments and their corresponding panels, the bioMérieux GP68 card necessitates confirmatory testing at a Minimum Inhibitory Concentration (MIC) of 2 µg/mL. This requirement is absent for panels from other manufacturers, as their MIC ranges adequately encompass all established breakpoints.

**Table 2:** Proportion of AST kit/mehod of *S. pneumoniae.*

AST Kit/method	Proportion(%)
Vitek GP68^a^	38.76
Disk diffusion	32.91
Gradient diffusion^b^	22.78
DL-96STREP/AST^c^	21.20
BD SMIC/ID-2^d^	8.07
TR-96或STR-AST^e^	4.91
DL-120STREP/AST^c^	4.22
Auto streptococcus AST^f^	2.34
Sensititre STP6F^g^	1.52
others	16.53

^a^bioMérieux company

^b^two brand including Auto and Biokont company

^c^DL Biotech company

^d^Becton, Dickinson and company

^e^Mindray company

^f^Autobio company

^g^Thermofisher company

#### Quality control for AST.

A significant proportion (44.10%, 1454/3297) did not perform routine QC for S. pneumoniae AST. Of these 1454 laboratories, 41.4% were from tertiary hospitals and 58.6% from secondary hospitals and others. For laboratories that performed routine QC, 63.65% were from tertiary hospitals and 36.35% from secondary hospitals and others. Among those not performing QC, 90.72% (1319/1454) lacked the specific QC strain.Only 49.65% (1637/3297) of all laboratories reported using ATCC 49619, the recommended QC strain for S. pneumoniae AST.

#### MALDI-TOF MS usage and QC.

30.15% (994/3297) of laboratories used MALDI-TOF MS for S. pneumoniae identification.Among these, 84.51%(840/994) were from tertiary hospitals and 15.49%(154/994) from secondary hospitals.Common brands: bioMérieux (37.32%), Bruker (24.14%), Autobio (21.23%).QC practices varied widely, with 13.38% relying solely on manufacturer-engineered calibration/QC.

This national survey provides a comprehensive overview of current laboratory practices for respiratory specimen processing and S. pneumoniae workup in CARSS hospitals across China.Our findings indicate that sputum remains the most frequently submitted specimen for bacterial culture (39.92%),a pattern consistent with previous reports from China where sputum is easily obtained and non-invasive.However, the over-reliance on sputum raises concerns about contamination and overdiagnosis, as lower respiratory tract specimens are often colonized by oropharyngeal flora.Encouragingly, a majority of laboratories perform routine Gram staining on sputum (71.55%) and BALF (66.88%), which is vital for initial assessment and guiding culture workup [8,16,17]. The reported criteria for sputum acceptability(>25 WBCs/LPF and <10 SECs/LPF) align well with established guidelines [18], suggesting good awareness of quality assessmen tamong most laboratories. Nevertheless, nearly half of the laboratories (49.83%) perform acceptability screening for BALF, and among those, 44.71% use the same criteria as for sputum—a practice that may not be optimal given the lower expected squamous epithelial cell content in BALF. This highlights an area for further guideline harmonization.

The identification of *S. pneumoniae* relies on a combination of traditional methods (Optochin/Bile Solubility testing), biochemistry-based automated systems, and modern techniques such as MALDI-TOF MS. The increasing adoption of MALDI-TOF MS (30.15%) represents a positive trend, as it enables rapid and accurate identification [19]. However, the adoption rate varies considerably by hospital level: tertiary hospitals reported a notably higher usage rate (84.51%) compared to secondary hospitals (15.49%), reflecting resource disparities.However, variations in calibration and quality control practices for MALDI-TOF MS highlight an area requiring standardization.Specifically, 13.38% of MALDI-TOF MS users relied solely on manufacturer-engineered calibration/QC without performing independent quality checks, and 18.14% did not follow supplementary identification procedures required by the manufacturer’s documentation. Given that some MALDI-TOF MS platforms cannot reliably distinguish S. pneumoniae from other *Streptococcus mitis* group species without additional tests (e.g., optochin susceptibility), this gap poses a risk of misidentification. Additionally, for the MALDI-TOF MS platforms used, it is essential to strictly adhere to the manufacturer's guidelines regarding necessary supplementary tests.

A concerning finding is the high percentage of laboratories (44.10%) not performing routine QC for S. pneumoniae AST, primarily due to the lack of the specific QC strain ATCC 49619. Reliable AST results are fundamental for guiding treatment and surveillance [20]. The absence of routine QC compromises the accuracy and reliability of susceptibility data, potentially impacting patient careand national resistance monitoring efforts through CARSS. Our data further reveal that among laboratories not performing QC, 90.72% explicitly cited the lack of the QC strain as the reason, suggesting that supply chain and cost barriers are major obstacles. This underscores an urgent need to promote and facilitate the consistent use of appropriate QC strains. Tertiary hospitals (65.28% performed routine QC) performed better than non-tertiary hospitals (44.17%), but both levels were suboptimal. This underscores an urgent need to promote and facilitate the consistent use of appropriate QC strains. National and regional public health authorities should consider subsidizing the distribution of ATCC 49619 or establishing a centralized QC strain supply network for CARSS member hospitals.

The diversity of antimicrobial susceptibility testing (AST) methods—including automated systems, disk diffusion, and gradient diffusion tests—reflects variations in resources and preferences across laboratories. Notably, the Vitek GP68 card requires confirmatory testing at an MIC of 2 µg/mL, but our survey did not assess adherence to this requirement; this represents a knowledge gap for future studies.While this methodological diversity can be managed with appropriate standardization and quality control (QC), the widespread lack of QC in a significant proportion of laboratories using these methods remains a serious concern.In fact, the QC deficit was observed across all AST method categories, not limited to any single platform. This suggests that the problem is systemic rather than technology-specific. In 2025, the Clinical and Laboratory Standards Institute (CLSI) introduced the concept of the Individualized Quality Control Plan (IQCP) for antimicrobial susceptibility testing (AST) in its M100 document (35th edition) [21]. This risk‑based framework allows laboratories to customize QC frequency and procedures based on their own risk assessments, offering a flexible alternative to traditional daily QC.Given that many laboratories face practical difficulties in obtaining routine QC strains—such as limited sources, long procurement cycles, and high costs—promoting the 2025 CLSI‑endorsed IQCP framework as a flexible and resource‑efficient alternative could be a pragmatic solution for those struggling with conventional QC strain acquisition.This study has limitations. The data are self-reported, which might introduce reporting bias. The survey captures practices at a specific point in time and may not reflect ongoing changes.

## Conclusions

This national survey reveals that while routine Gram staining and sputum acceptability criteria are generally aligned with guidelines, major gaps persist in AST quality control for S. pneumoniae. Nearly half of CARSS laboratories perform no routine QC, and fewer than 50% use the recommended ATCC 49619 strain. Immediate actions are needed to mandate QC protocols, ensure supply of QC strains, and strengthen laboratory training. Addressing these deficiencies is essential for reliable resistance surveillance and optimal patient management in China

## Supporting information

S1 File S1 questionnaire. (XLSX)
