# Supplementary material for: A national survey on laboratory practices for specimen processing, streptococcus pneumoniae identification and antimicrobial susceptibility testing in CARSS Hospitals in China
Source: PLoS One. 2026 Aug 12;21(8):e0353484. doi: 10.1371/journal.pone.0353484 (PMC13465804; doi:10.1371/journal.pone.0353484)
Supplement: Supplementary file 1 [file pone.0353484.s002.docx]

Dear Editor,

Thank you for your letter and for the opportunity to revise our submission. We have carefully addressed each of the points you raised. Our responses are detailed below, and we have made the corresponding changes to the manuscript and the online submission system.

**Point 1:** The dataset contains text or data not in English; please provide an English‑language version.

**Response:**

We have now translated the entire dataset into English. The English‑version data file has been uploaded as Supporting Information (see file “S1_Data_English.csv” [or whatever your file name is]) and replaces the previous non‑English version. We confirm that the dataset is now fully in English, which we trust will facilitate evaluation and compliance with PLOS’s Data Availability policy.

**Point 2:** Please clarify the type of informed consent obtained from participants (written or verbal, and if verbal, how it was documented and witnessed).

**Response:**

We have added a clear statement in the Methods section, under the Ethics statement subsection, specifying the type of informed consent obtained. Specifically, we inserted the following sentence directly in the revised manuscript:

“This survey was based on the CARSS data reporting system. All participants gave their informed consent before completing and submitting the questionnaire.”

We are confident that these revisions have substantially improved the manuscript. We thank the editor and reviewers for their thoughtful and constructive feedback, which has made our work stronger.

We look forward to your positive decision.

Sincerely,
