# Supplementary material for: A national survey on laboratory practices for specimen processing, streptococcus pneumoniae identification and antimicrobial susceptibility testing in CARSS Hospitals in China
Source: PLoS One. 2026 Aug 12;21(8):e0353484. doi: 10.1371/journal.pone.0353484 (PMC13465804; doi:10.1371/journal.pone.0353484)
Supplement: Peer review history

# Peer review: Decision Letter 0

**DOI:** 10.1371/journal.pone.0353484.r001

**Authors:** Benjamin M. Liu

18 Mar 2026

Dear Dr. Jian,

Thank you for submitting your manuscript to PLOS ONE. After careful consideration, we feel that it has merit but does not fully meet PLOS ONE’s publication criteria as it currently stands. Therefore, we invite you to submit a revised version of the manuscript that addresses the points raised during the review process.

**ACADEMIC EDITOR'S COMMENTS:**

Please submit your revised manuscript by May 1 2026. If you will need more time than this to complete your revisions, please reply to this message or contact the journal office at plosone@plos.org. When you're ready to submit your revision, log on to https://www.editorialmanager.com/pone/ and select the 'Submissions Needing Revision' folder to locate your manuscript file.

- A letter that responds to each point raised by the academic editor and reviewer(s). You should upload this letter as a separate file labeled 'Response to Reviewers'.
- A marked-up copy of your manuscript that highlights changes made to the original version. You should upload this as a separate file labeled 'Revised Manuscript with Track Changes'.
- An unmarked version of your revised paper without tracked changes. You should upload this as a separate file labeled 'Manuscript'.

If you would like to make changes to your financial disclosure, please include your updated statement in your cover letter. Guidelines for resubmitting your figure files are available below the reviewer comments at the end of this letter.

If applicable, we recommend that you deposit your laboratory protocols in protocols.io to enhance the reproducibility of your results. Protocols.io assigns your protocol its own identifier (DOI) so that it can be cited independently in the future. For instructions see: https://journals.plos.org/plosone/s/submission-guidelines#loc-laboratory-protocols. Additionally, PLOS ONE offers an option for publishing peer-reviewed Lab Protocol articles, which describe protocols hosted on protocols.io. Read more information on sharing protocols at https://plos.org/protocols?utm_medium=editorial-email&utm_source=authorletters&utm_campaign=protocols.

We look forward to receiving your revised manuscript.

Kind regards,

Benjamin M. Liu, MBBS, PhD, D(ABMM), MB(ASCP)

Academic Editor

PLOS One

Journal requirements:

When submitting your revision, we need you to address these additional requirements.

1. Please ensure that your manuscript meets PLOS ONE's style requirements, including those for file naming. The PLOS ONE style templates can be found at

https://journals.plos.org/plosone/s/file?id=wjVg/PLOSOne_formatting_sample_main_body.pdf and

https://journals.plos.org/plosone/s/file?id=ba62/PLOSOne_formatting_sample_title_authors_affiliations.pdf

2. Please provide additional details regarding participant consent. In the ethics statement in the Methods and online submission information, please ensure that you have specified (1) whether consent was informed and (2) what type you obtained (for instance, written or verbal, and if verbal, how it was documented and witnessed). If your study included minors, state whether you obtained consent from parents or guardians. If the need for consent was waived by the ethics committee, please include this information.

If you are reporting a retrospective study of medical records or archived samples, please ensure that you have discussed whether all data were fully anonymized before you accessed them and/or whether the IRB or ethics committee waived the requirement for informed consent. If patients provided informed written consent to have data from their medical records used in research, please include this information.

3. Please include a separate caption for each figure in your manuscript.

4. We note that Figure 1 in your submission contain [map/satellite] images which may be copyrighted. All PLOS content is published under the Creative Commons Attribution License (CC BY 4.0), which means that the manuscript, images, and Supporting Information files will be freely available online, and any third party is permitted to access, download, copy, distribute, and use these materials in any way, even commercially, with proper attribution. For these reasons, we cannot publish previously copyrighted maps or satellite images created using proprietary data, such as Google software (Google Maps, Street View, and Earth). For more information, see our copyright guidelines: http://journals.plos.org/plosone/s/licenses-and-copyright.

We require you to either (1) present written permission from the copyright holder to publish these figures specifically under the CC BY 4.0 license, or (2) remove the figures from your submission:

1. You may seek permission from the original copyright holder of Figure 1 to publish the content specifically under the CC BY 4.0 license.

We recommend that you contact the original copyright holder with the Content Permission Form (http://journals.plos.org/plosone/s/file?id=7c09/content-permission-form.pdf) and the following text:

“I request permission for the open-access journal PLOS ONE to publish XXX under the Creative Commons Attribution License (CCAL) CC BY 4.0 (http://creativecommons.org/licenses/by/4.0/). Please be aware that this license allows unrestricted use and distribution, even commercially, by third parties. Please reply and provide explicit written permission to publish XXX under a CC BY license and complete the attached form.”

Please upload the completed Content Permission Form or other proof of granted permissions as an "Other" file with your submission.

In the figure caption of the copyrighted figure, please include the following text: “Reprinted from [ref] under a CC BY license, with permission from [name of publisher], original copyright [original copyright year].”

2. If you are unable to obtain permission from the original copyright holder to publish these figures under the CC BY 4.0 license or if the copyright holder’s requirements are incompatible with the CC BY 4.0 license, please either i) remove the figure or ii) supply a replacement figure that complies with the CC BY 4.0 license. Please check copyright information on all replacement figures and update the figure caption with source information. If applicable, please specify in the figure caption text when a figure is similar but not identical to the original image and is therefore for illustrative purposes only.

The following resources for replacing copyrighted map figures may be helpful:

USGS National Map Viewer (public domain): http://viewer.nationalmap.gov/viewer/

The Gateway to Astronaut Photography of Earth (public domain): http://eol.jsc.nasa.gov/sseop/clickmap/

Maps at the CIA (public domain): https://www.cia.gov/library/publications/the-world-factbook/index.html and https://www.cia.gov/library/publications/cia-maps-publications/index.html

NASA Earth Observatory (public domain): http://earthobservatory.nasa.gov/

Landsat: http://landsat.visibleearth.nasa.gov/

USGS EROS (Earth Resources Observatory and Science (EROS) Center) (public domain): http://eros.usgs.gov/#

Natural Earth (public domain): http://www.naturalearthdata.com/

5. We note that you have indicated that there are restrictions to data sharing for this study. For studies involving human research participant data or other sensitive data, we encourage authors to share de-identified or anonymized data. However, when data cannot be publicly shared for ethical reasons, we allow authors to make their data sets available upon request. For information on unacceptable data access restrictions, please see http://journals.plos.org/plosone/s/data-availability#loc-unacceptable-data-access-restrictions.

Before we proceed with your manuscript, please address the following prompts:

a) If there are ethical or legal restrictions on sharing a de-identified data set, please explain them in detail (e.g., data contain potentially identifying or sensitive patient information, data are owned by a third-party organization, etc.) and who has imposed them (e.g., a Research Ethics Committee or Institutional Review Board, etc.). Please also provide contact information for a data access committee, ethics committee, or other institutional body to which data requests may be sent.

b) If there are no restrictions, please upload the minimal anonymized data set necessary to replicate your study findings to a stable, public repository and provide us with the relevant URLs, DOIs, or accession numbers. Please see http://www.bmj.com/content/340/bmj.c181.long for guidelines on how to de-identify and prepare clinical data for publication. For a list of recommended repositories, please see https://journals.plos.org/plosone/s/recommended-repositories. You also have the option of uploading the data as Supporting Information files, but we would recommend depositing data directly to a data repository if possible.

Please update your Data Availability statement in the submission form accordingly.

If the reviewer comments include a recommendation to cite specific previously published works, please review and evaluate these publications to determine whether they are relevant and should be cited. There is no requirement to cite these works unless the editor has indicated otherwise.

[Note: HTML markup is below. Please do not edit.]

Reviewers' comments:

Reviewer's Responses to Questions

**Comments to the Author**

1. Is the manuscript technically sound, and do the data support the conclusions?

Reviewer #1: Yes

Reviewer #2: Yes

Reviewer #3: No

**********

2. Has the statistical analysis been performed appropriately and rigorously?

Reviewer #1: Yes

Reviewer #2: Yes

Reviewer #3: I Don't Know

**********

3. Have the authors made all data underlying the findings in their manuscript fully available?

The PLOS Data policy

Reviewer #1: Yes

Reviewer #2: Yes

Reviewer #3: Yes

**********

4. Is the manuscript presented in an intelligible fashion and written in standard English?

Reviewer #1: Yes

Reviewer #2: Yes

Reviewer #3: Yes

**********

Reviewer #1: Everything is fine and us according to ethics and is relevant and is applied appropriately .application of stats is alright and relevant and survey is taken with appropriate method and is relevant for future applicability

Reviewer #2: The manuscript entitled A National Survey on Laboratory Practices for Specimen Processing, Streptococcus

pneumoniae Identification and Antimicrobial Susceptibility Testing in CARSS Hospitals in China is well structured and written, I would suggest using advanced statical analysis tool for the significance especially p-value, also I would suggest to elaborate the discussion part. Conclusion need to be crisp.

Figure legends are not given.

In figure 2 (f) is missing for the last figure.

Reviewer #3: This manuscript reports findings from a nationwide survey evaluating laboratory practices for respiratory specimen processing of Streptococcus pneumoniae. The topic is highly relevant to antimicrobial resistance (AMR) surveillance and laboratory standardization. The large sample size is a major strength. However, the manuscript would benefit from clearer articulation of the study objectives and a more explicit explanation of how the findings will contribute to the research community.

Could the authors clarify whether the objective was to assess guideline compliance, benchmark laboratory practices, or evaluate variability across hospital levels?

While the dataset is valuable, the manuscript would benefit from a clearer research focus and analytical depth. Currently, the presentation is largely descriptive, and incorporating defined research questions, comparisons, or inferential analyses would strengthen its positioning as a research article.

**********

what does this mean?). If published, this will include your full peer review and any attached files.

If you choose “no”, your identity will remain anonymous but your review may still be made public.

**Do you want your identity to be public for this peer review?**  For information about this choice, including consent withdrawal, please see our Privacy Policy

Reviewer #1: **Yes:**  Kunal Joon

Reviewer #2: **Yes:**  Dr. Asiya Khan

Reviewer #3: No

**********

[NOTE: If reviewer comments were submitted as an attachment file, they will be attached to this email and accessible via the submission site. Please log into your account, locate the manuscript record, and check for the action link "View Attachments". If this link does not appear, there are no attachment files.]

To ensure your figures meet our technical requirements, please review our figure guidelines: https://journals.plos.org/plosone/s/figures

You may also use PLOS’s free figure tool, NAAS, to help you prepare publication quality figures: https://journals.plos.org/plosone/s/figures#loc-tools-for-figure-preparation.

NAAS will assess whether your figures meet our technical requirements by comparing each figure against our figure specifications.

# Author comment: Author response to Decision Letter 1

**DOI:** 10.1371/journal.pone.0353484.r002

22 Jun 2026

Responses to Reviewer #1

Comment: Everything is fine and is according to ethics and is relevant and is applied appropriately. Application of stats is alright and relevant and survey is taken with appropriate method and is relevant for future applicability.

Response: We sincerely thank Reviewer #1 for the positive assessment and encouraging words. We are glad that the methodology, statistical application, and ethical aspects meet the reviewer’s expectations. No further changes were requested, but we have carefully re‑checked the manuscript for any minor improvements.

Responses to Reviewer #2

Comment 1: The manuscript entitled “A National Survey on Laboratory Practices for Specimen Processing, Streptococcus pneumoniae Identification and Antimicrobial Susceptibility Testing in CARSS Hospitals in China” is well structured and written. I would suggest using advanced statistical analysis tool for the significance especially p-value, also I would suggest to elaborate the discussion part. Conclusion need to be crisp. Figure legends are not given. In figure 2 (f) is missing for the last figure.

Response:We greatly appreciate the reviewer’s constructive suggestions. We have addressed each point as follows:

Point 1 (Advanced statistical analysis and p-values):Our study is a descriptive cross-sectional survey without predefined comparative hypotheses or experimental groups. The primary aim was to report current practices across CARSS hospitals using frequencies and proportions. Therefore, inferential statistics (e.g., p-values) were not applicable. However, we acknowledge the reviewer’s suggestion and have added the following clarification in the Methods section (Data Analysis subsection) to justify our descriptive approach:

“As this was an exploratory national survey without a priori hypotheses or comparator groups, only descriptive statistics (counts, percentages, averages, and ranges) were used. Inferential statistics were not performed because the study aimed to characterize the current landscape rather than test for differences between subgroups.”

Point 2 (Elaborate the Discussion section):

We have substantially expanded the Discussion section to provide deeper interpretation, comparisons with previous studies, and practical implications. Summary of changes:

Added hospital-level stratification (tertiary vs. secondary) for MALDI-TOF MS usage and QC performance.

Expanded discussion of MALDI-TOF MS QC gaps (specific percentages, risk of misidentification, manufacturer guidelines).

Deepened analysis of AST QC deficit (reasons for lack of QC strain, tertiary vs. non-tertiary differences, supply chain barriers).

Discussed Vitek GP68 confirmatory testing requirement as a knowledge gap.

Introduced IQCP and suggested it as a pragmatic solution.

Strengthened actionable recommendations (subsidized QC strain distribution, national supply network).

Point 3 (Conclusion needs to be crisp):

We have rewritten the Conclusion to be more concise and impactful. The new version is:

“This national survey reveals that while routine Gram staining and sputum acceptability criteria are generally aligned with guidelines, major gaps persist in AST quality control for S. pneumoniae. Nearly half of CARSS laboratories perform no routine QC, and fewer than 50% use the recommended ATCC 49619 strain. Immediate actions are needed to mandate QC protocols, ensure supply of QC strains, and strengthen laboratory training. Addressing these deficiencies is essential for reliable resistance surveillance and optimal patient management in China.”

Point 4 (Figure legends missing):

We have added comprehensive figure legends for all figures. For Figure 1, the legend states: *“Geographical distribution of survey questionnaires across provinces in China (N=3,297). Numbers indicate the count of responses from each province.”* For Figure 2, the legend now reads: *“Proportional distribution of different specimen types among annual bacterial culture submissions (N=3,297). (a) Sputum, (b) Blood, (c) Urine, (d) Bronchoalveolar lavage fluid (BALF), (e) Others.

Point 5 (Figure 2 missing panel (f)):

Thank you for your careful observation. We apologize for the labeling error in Figure 2 of the original submission. Due to a formatting mistake, panel (d) “Bronchoalveolar lavage fluid (BALF)” was inadvertently duplicated and labeled as panel (e) “Other specimen types,” while what was originally shown as panel (f) was actually the correct panel for “Other specimen types.” Consequently, there was no genuine panel (f). We have now corrected Figure 2 so that the five specimen types are accurately labeled as (a) Sputum, (b) Blood, (c) Urine, (d) BALF, and (e) Others. The revised figure is included in the resubmitted manuscript. We appreciate your understanding.

Response to Reviewer #3

Reviewer #3: This manuscript reports findings from a nationwide survey evaluating laboratory practices for respiratory specimen processing of Streptococcus pneumoniae. The topic is highly relevant to antimicrobial resistance (AMR) surveillance and laboratory standardization. The large sample size is a major strength. However, the manuscript would benefit from clearer articulation of the study objectives and a more explicit explanation of how the findings will contribute to the research community.

Could the authors clarify whether the objective was to assess guideline compliance, benchmark laboratory practices, or evaluate variability across hospital levels?

While the dataset is valuable, the manuscript would benefit from a clearer research focus and analytical depth. Currently, the presentation is largely descriptive, and incorporating defined research questions, comparisons, or inferential analyses would strengthen its positioning as a research article.

Response: We thank Reviewer #3 for recognizing the relevance and strength of our large dataset. We fully agree that the study objectives need to be more explicit. In response, we have made the following revisions:

1. Clarification of study objectives in the Introduction (end of Introduction section):

We have rewritten the final paragraph of the Introduction to clearly state the three specific objectives:

*“This national survey aimed to: (1) assess compliance with existing national and international guidelines for respiratory specimen processing and S. pneumoniae identification/AST; (2) benchmark current laboratory practices across CARSS hospitals to identify gaps and variability; and (3) provide evidence-based recommendations to standardize procedures, particularly for AST quality control. While we primarily describe the current landscape, the data also allow comparisons with recommended standards and highlight areas requiring urgent intervention.”*

2. Explanation of contribution to the research community:

We have added a new paragraph at the end of the Introduction (before Methods) to explicitly state how the findings will benefit the research community:

*“The findings of this survey will serve as a critical baseline for future interventions and policy-making. By identifying specific deficiencies—such as the widespread lack of routine AST QC and the underutilization of the recommended QC strain ATCC 49619—this study provides actionable targets for quality improvement programs. Furthermore, the data inform the CARSS network on the current state of laboratory readiness, enabling targeted training and resource allocation. The methodological framework can also be adapted for similar surveys in other low- and middle-income countries.

3. Enhanced analytical depth (Discussion):

Although our study was designed as a descriptive survey, we have added comparative elements in the Discussion to increase analytical depth. Specifically:

We stratified key findings by hospital level (tertiary vs. non-tertiary) where data were available and added a brief sub-analysis in the Results. For example: *“Tertiary hospitals showed higher rates of routine QC performance (65.28%) compared to non-tertiary hospitals (44.17%), though both were suboptimal.”* This addresses the reviewer’s suggestion about variability across hospital levels.

We added inferential statements (without formal p-values, consistent with our descriptive approach) to highlight meaningful differences, e.g., *“A notably higher proportion of tertiary hospitals used MALDI-TOF MS (84.51%) compared to secondary hospitals (15.49%).

4. Explicit research questions:

We have added three explicit research questions at the end of the Introduction:

*“Specifically, we sought to answer: (1) What are the current specimen type distributions and sputum/BALF processing practices in CARSS hospitals? (2) To what extent do laboratories adhere to recommended methods for S. pneumoniae identification and AST? (3) What proportion of laboratories perform routine AST quality control, and which QC strains are used?”*

We believe these revisions substantially clarify the study’s focus and enhance its value to the research community.

Additional Revisions (Not Specifically Requested but Improved)

Language and grammar: We have carefully proofread the entire manuscript and corrected minor typographical errors (e.g., repeated word “laboratories” in the abstract, missing spaces).

Reference formatting: All references have been checked and formatted consistently according to PLOS ONE style.

Data availability statement: We have added a statement that the anonymized survey data are available from the corresponding author upon reasonable request, pending institutional approval.

We are confident that these revisions have substantially improved the manuscript. We thank the editor and reviewers for their thoughtful and constructive feedback, which has made our work stronger.

We look forward to your positive decision.

Sincerely,

Attachment Submitted filename: Response to Reviewers202606.docx

# Editor report: Decision Letter 1

**DOI:** 10.1371/journal.pone.0353484.r003

**Authors:** Benjamin M. Liu

25 Jun 2026

A National Survey on Laboratory Practices for Specimen Processing, Streptococcus pneumoniae Identification and Antimicrobial Susceptibility Testing in CARSS Hospitals in China

PONE-D-26-06099R1

Dear Dr. Jian,

We’re pleased to inform you that your manuscript has been judged scientifically suitable for publication and will be formally accepted for publication once it meets all outstanding technical requirements.

Within one week, you’ll receive an e-mail detailing the required amendments. When these have been addressed, you’ll receive a formal acceptance letter and your manuscript will be scheduled for publication.

An invoice will be generated when your article is formally accepted. Please note, if your institution has a publishing partnership with PLOS and your article meets the relevant criteria, all or part of your publication costs will be covered. Please make sure your user information is up-to-date by logging into Editorial Manager at Editorial Manager® and clicking the ‘Update My Information' link at the top of the page. For questions related to billing, please contact billing support.

If your institution or institutions have a press office, please notify them about your upcoming paper to help maximize its impact. If they’ll be preparing press materials, please inform our press team as soon as possible -- no later than 48 hours after receiving the formal acceptance. Your manuscript will remain under strict press embargo until 2 pm Eastern Time on the date of publication. For more information, please contact onepress@plos.org.

Kind regards,

Benjamin M. Liu, PhD, D(ABMM), MB(ASCP)

Academic Editor

PLOS One

Additional Editor Comments (optional):

Reviewers' comments:

# Editor report: Acceptance letter

**DOI:** 10.1371/journal.pone.0353484.r004

**Authors:** Benjamin M. Liu

PONE-D-26-06099R1

PLOS One

Dear Dr. Jian,

I'm pleased to inform you that your manuscript has been deemed suitable for publication in PLOS One. Congratulations! Your manuscript is now being handed over to our production team.

At this stage, our production department will prepare your paper for publication. This includes ensuring the following:

* All references, tables, and figures are properly cited

* All relevant supporting information is included in the manuscript submission,

* There are no issues that prevent the paper from being properly typeset

You will receive further instructions from the production team, including instructions on how to review your proof when it is ready. Please keep in mind that we are working through a large volume of accepted articles, so please give us a few days to review your paper and let you know the next and final steps.

Lastly, if your institution or institutions have a press office, please let them know about your upcoming paper now to help maximize its impact. If they'll be preparing press materials, please inform our press team within the next 48 hours. Your manuscript will remain under strict press embargo until 2 pm Eastern Time on the date of publication. For more information, please contact onepress@plos.org.

You will receive an invoice from PLOS for your publication fee after your manuscript has reached the completed accept phase. If you receive an email requesting payment before acceptance or for any other service, this may be a phishing scheme. Learn how to identify phishing emails and protect your accounts at https://explore.plos.org/phishing.

If we can help with anything else, please email us at customercare@plos.org.

Thank you for submitting your work to PLOS ONE and supporting open access.

Kind regards,

PLOS ONE Editorial Office Staff

on behalf of

Dr. Benjamin M. Liu

Academic Editor

PLOS One
